# Using Qualitative Methods to Explore Lay Explanatory Models, Health-Seeking Behaviours and Self-Care Practices of Podoconiosis Patients in North-West Ethiopia

**DOI:** 10.1371/journal.pntd.0004878

**Published:** 2016-08-18

**Authors:** Harrison S. Banks, Girmay Tsegay, Moges Wubie, Abreham Tamiru, Gail Davey, Max Cooper

**Affiliations:** 1 Brighton and Sussex Medical School, Brighton, United Kingdom; 2 Debre Markos University, Debre Markos, Ethiopia; 3 IOCC Podoconiosis Project, Debre Markos, Ethiopia; Michigan State University, UNITED STATES

## Abstract

**Background:**

Podoconiosis (endemic non-filarial elephantiasis) is a chronic, non-infectious disease resulting from exposure of bare feet to red-clay soil in tropical highlands. This study examined lay beliefs about three under-researched aspects of podoconiosis patients’ care: explanatory models, health-seeking behaviours and self-care.

**Methods:**

In-depth interviews and focus group discussions were undertaken with 34 participants (19 male, 15 female) between April-May 2015 at podoconiosis treatment centres across East and West Gojjam regions in north-west Ethiopia.

**Results:**

Explanatory models for podoconiosis included contamination from blood, magic, soil or affected individuals. Belief in heredity or divine punishment often delayed clinic attendance. All participants had tried holy water treatment and some, holy soil. Herbal treatments were considered ineffectual, costly and appeared to promote fluid escape. Motivators for clinic attendance were failure of traditional treatments and severe or disabling symptoms. Patients did not report self-treatment with antibiotics. Self-care was hindered by water being unavailable or expensive and patient fatigue.

**Conclusion:**

A pluralistic approach to podoconiosis self-treatment was discovered. Holy water is widely valued, though some patients prefer holy soil. Priests and traditional healers could help promote self-care and “signpost” patients to clinics. Change in behaviour and improving water access is key to self-care.

## Author Summary

Podoconiosis is a disease that is caused by long-term exposure to red clay soil found in tropical highland areas. This causes swelling of the legs, episodes of intense pain and severe disability. Sufferers of the condition experience stigma and exclusion from the community. Previous work has demonstrated a low rate of re-attendance to clinics and that many patients fail to adhere to their treatment regime of improving foot hygiene. The present study explored areas of health-seeking behaviour and self-care practices to discover the reason behind these failures. It was found that explanatory models of disease causation had a significant impact on decisions to seek healthcare and that participants only turned to Western medical clinics after failure of traditional treatments and with particularly severe symptoms. We identified several form of traditional treatment for the disease, and these tended to be based on cleansing, fluid extraction or faith/symbolism. The most important barrier to self-care and adhering to treatment regimens was an inability to collect adequate water. We call for greater integration with traditional healers, improved access to water through collaboration with other NGOs and the government and the use of expert patients to disseminate information and signpost patients to clinics.

## Introduction

Podoconiosis (endemic non-filiarial elephantiasis) is a chronic non-infectious disease affecting individuals whose feet have been exposed to red clay soil found in the tropical highland areas.[1] A very recent study from Molla et al. demonstrates the importance of phyllosilicate clay minerals, particularly smectite, mica groups and quartz (crystalline silica) in the pathogenesis of podoconiosis.[2] A strong genetic element has been identified, following an autosomal co-dominant major gene pattern,[3] with identified variation in the human leukocyte antigen (HLA) class II locus increasing risk by 2 to 3 times.[4]

Those most commonly affected are lower socioeconomic groups, who often struggle to afford shoes, socks or water to wash with. Worldwide, an estimated 4million people live with podoconiosis.[1] A particularly high prevalence has been documented in highland areas of East and Central Africa.[5–7] The prevalence in Ethiopia is 4%, with increasing prevalence at older ages.[8] Approximately 1million people are affected by the disease in Ethiopia.[1]

Podoconiosis is characterised by a prodromal phase consisting of itching and burning sensations in the forefoot and lower leg. Early changes observed include plantar oedema, splaying of the toes, hyperkeratosis with the formation of moss-like papillomata and rigid toes.[1] Long-standing disease is associated with fusion of interdigital spaces and ankylosis of interphalangeal or ankle joints.[1] On average, five times per year, patients suffer from episodes of acute adenolymphangitis that are characterised by pyrexia and intense pain, often necessitating days off work.[1]

It is estimated that podoconiosis patients lose 45% of total working days per year.[9] In one Ethiopian zone of 1.5million people, podoconiosis is estimated to cost over US$16million per year.[9] Stigmatisation against podoconiosis patients is common,[10] with patients being excluded from school, churches, mosques, and barred from marriage with unaffected individuals.[11] Given this background, the discovery of high levels of mental distress and overall lower quality of life amongst podoconiosis patients is unsurprising.[12,13]

Primary prevention consists of the use of footwear. Secondary prevention involves foot hygiene (washing daily with water and soap, and using antiseptics and emollients), with compression bandaging to reduce soft swelling. A small uncontrolled clinical evaluation from Sikorski et al. demonstrated that clinical improvements can be achieved if these simple measures are strictly adhered to.[14] In 2010, International Orthodox Christian Charities (IOCC, an international non-governmental organisation) started a programme aiming to prevent and treat podoconiosis in East and West Gojjam zones in the Amhara region of Ethiopia.

Several studies have identified significant barriers to podoconiosis patients’ attendance at IOCC clinics, with major challenges being: geographical isolation, cost, domestic duties and stigma.[15,16] It was hypothesised that additional factors may influence peoples’ health-seeking behaviour, especially early in their illness. Early health-seeking behaviour models were based on pathways to care, in which a stepwise journey starts at identification of symptoms and ends with the use of care.[17] More recent research in this field has moved from pre-defined trajectories and has largely sought to capture wider determinants of health-seeking behaviour. For example, Andersen’s model which groups factors that influence utilisation into three main categories: environmental, predisposing/enabling and health system.[18] This was due to the criticism that health-seeking behaviour research places excessive emphasis on individual choice and an assumption that individuals are autonomous in decision making.[19] We therefore adopted a dynamic notion of health-seeking behaviour where perceived eligibility to receive care represents *“a continually negotiated property of individuals*, *subject to multiple influences arising both from people and their social contexts and from macro-level influences on allocation of resources and configuration of services”*.[20] Health-seeking behaviour, therefore, is ultimately determined by the interaction between the individual, their socioeconomic situation and the agents of available treatment services, i.e. not a pre-defined trajectory. An additional dimension of understanding health-seeking behaviour in early stage podoconiosis was to explore explanatory models and their influence on treatment choice and compliance with such treatment. Previous studies highlight that podoconiosis patients fail to comply with daily washing treatment regimes.[21] Poor adherence to treatment regimes in relation to chronic conditions is associated with increased rates of complications and higher treatment costs.[22] Effective communication around conflicting explanatory models is shown to be a *“major determinant of patient compliance*, *satisfaction and appropriate use of medical facilities”*.[23] The present study, thus, also aimed to explore explanatory models and beliefs about and barriers to self-care of amongst podoconiosis patients.

## Methods

### Study setting

**Fig 1:**
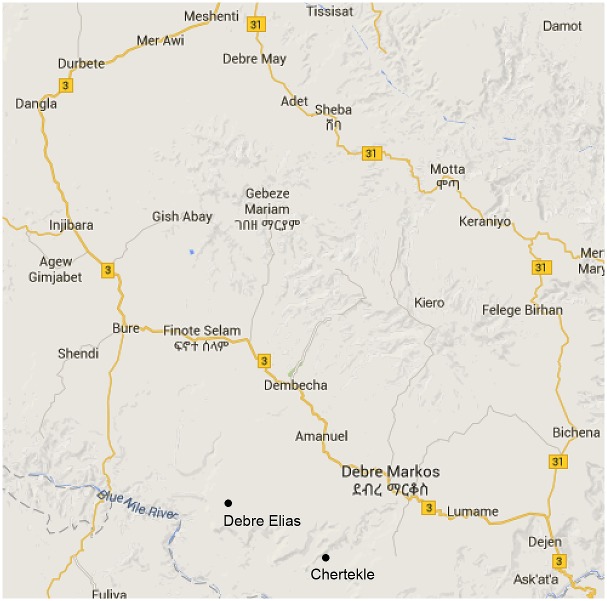
Area map for the present study. Participants were enrolled from treatment sites at Dembecha, Chertekle, Fenote Selam, Debre Elias, Amanule and Bure. Map was created using Google Maps and Microsoft Paint.

Amhara is the second largest region in Ethiopia with a 2007 population of 17,214,056; of whom only 12.27% resided in urban areas.[24] The majority of inhabitants in the Gojjam area are agricultural workers, only 19.5% in West Gojjam and 11.4% in East Gojjam reported as being in non-farm related occupations.[24] The soil and climatic conditions that induce podoconiosis, in particular high altitude (>1000m), seasonal rainfall (over 1000mm annually) and the presence of red-clay soil,[25] are all present in Amhara region, especially East and West Gojjam zones. Participants for the present study were enrolled from treatment sites in these zones at Dembecha, Chertekle, Fenote Selam, Debre Elias, Amanuel and Bure (see Fig 1 for a map of the study area).

### Study design

A cross-sectional exploratory study was conducted between April-May 2015 at IOCC treatment centres across East and West Gojjam zones in North-West Ethiopia (Fig 1). Eleven IDIs, three FGDs and two key-informant interviews (KIIs) were conducted. Convenience sampling was used in order to recruit participants.

Interviews were conducted in Amharic by a bi-lingual researcher experienced in conducting qualitative research. A semi-structured interview guide was developed to encourage podoconiosis patients to discuss their understanding[23] and recognition of their illness,[20] when they sought help and who this was from, any traditional treatments tried, their motivations for attending IOCC services were and beliefs about adherence to self-care regimes. Questions were designed to be open and the interviewer given freedom to explore respondents’ responses, to elucidate deeper understanding and to identify themes not anticipated by the research team. Questions were translated into Amharic, piloted among local staff and then modified to verify cultural meanings before use with participants. As particular themes emerged, interviews were modified to include questions to explore these themes more fully. FGD topics were created following themes that emerged from the IDIs.

Current IOCC patients were interviewed on the day of their attendance at clinic. For past IOCC users and non-IOCC patient’s, a mutually convenient time was agreed on when to conduct the IDI. IDIs were conducted in a private setting for up to 58 minutes. FGDs also took place at treatment centres in a private setting. These took up to 2 hour 10 minutes and all included both male and female participants who were all affected by podoconiosis. Interviews were recorded, transcribed, translated and entered into Microsoft Word to facilitate data coding, text-searching and analysis. Data collection continued until no new themes emerged, i.e. until data saturation was reached.

### Data analysis

Following transcription, data were organised using manual coding techniques to categorise and generate themes. The method for generating codes involved using an integrated approach to developing code structure. This process involves both inductive development of codes as well as a deductive organising framework for code types.[26] Validity was promoted through having two independent coders organise the data. The coders resolved issues and incorporated new themes through regular meetings and discussion.

### Ethical issues

Study participants were only recruited once they were fully aware of the purpose of the research and the methods of data collection. Since many participants were illiterate, individual consent was requested orally and signed by an Amharic-speaking witness, once the participant had read and/or discussed the participant information sheet. Participants were informed that they had a right to stop or skip questions during the interview at any time, and that if they changed their mind about being part of the study they could contact the IOCC and their data would be removed. Participants were given unique identification numbers to maintain confidentiality and audio recordings were destroyed following anonymized transcription. In the results below, quotations are identified as follows: *[F2*,*2]* indicates that the participant was labelled as participant 2 from FGD 2; [8] is participant 8 from the IDIs, *[K1]* was the first key-informant interview.

## Results

### Characteristics of study population

A total of 34 participants were interviewed, comprising the following: past and present IOCC patients (13 male, 12 female), non-IOCC patients (2 female), patient association leaders (4 male, 1 female) and health workers (2 male). The age range of participants was 26–90 years. Most participants were subsistence farmers or daily labourers, rural dwellers, married and unable to read and/or write. See Table 1 for study population characteristics and assigned participant codes.

**Table 1:** Study population characteristics and assigned participant codes.

Participant Code	Area	Age	Gender	Employment	Treatment Status
**1.**	Chertekle	31	M	Farmer	Current patient
**2.**	Chertekle	55	M	Priest	Former patient
**3.**	Chertekle	48	M	Farmer	Current patient
**4.**	Chertekle	26	F	Merchant	Current patient
**5.**	Dembecha	70	M	Farmer	Former patient
**6.**	Finote-selam	30	F	Farmer	Current patient
**7.**	Finote-selam	30	F	Farmer	Current patient
**8.**	Amanuel	33	M	Farmer	Former patient
**9.**	Chertekle	67	M	Farmer	Current Patient
**10.**	Dangla	70	F	Daily Labourer	Non-patient
**11.**	Dangla	40	F	Daily Labourer	Non-patient
**F1,1**	Finote-selam	51	M	Farmer	Current patient
**F1,2**	Finote-selam	55	M	Farmer	Current patient
**F1,3**	Finote-selam	46	M	Daily Labourer	Current patient
**F1,4**	Finote-selam	36	F	Daily Labourer	Current patient
**F1,5**	Finote-selam	43	F	Daily Labourer	Current patient
**F1,6**	Finote-selam	58	F	Daily Labourer	Current patient
**F1,7**	Finote-selam	30	F	Daily Labourer	Current patient
**F2,1**	Debre Elias	51	M	PA Leader/Farmer	Former patient
**F2,2**	Debre Elias	54	M	PA Leader/Farmer	Former patient
**F2,3**	Debre Elias	50	F	PA Leader/Farmer	Former patient
**F2,4**	Debre Elias	38	M	Farmer	Current patient
**F2,5**	Debre Elias	45	M	PA Leader/Farmer	Former patient
**F2,6**	Debre Elias	47	M	PA Leader/Farmer	Current patient
**F3,1**	Bure	45	F	Farmer	Current patient
**F3,2**	Bure	45	F	Farmer	Current patient
**F3,3**	Bure	40	F	Farmer	Current patient
**F3,4**	Bure	50	F	Farmer	Current patient
**F3,5**	Bure	54	M	Farmer	Current patient
**F3,6**	Bure	90	M	Farmer	Current patient
**F3,7**	Bure	73	M	Farmer	Current patient
**F3,8**	Bure	36	M	Farmer	Current patient
**K1**	Debre Markos	32	M	Health worker	N/A
**K2**	Debre Markos	29	M	Health worker	N/A

PA—Patient Association; 1 –In-depth interview participant 1; F1,5 –Focus group 1 participant 5; K1 –Key informant 1; Current patient—currently receiving treatment from IOCC clinic; former patient—has previously received treatment from IOCC clinic; non-patient—has never received treatment from IOCC clinic.

### Health beliefs

#### Recognising podoconiosis

Podoconiosis was described as a gradual onset of swelling that started in the toes and spread up the legs and considered distinct from other diseases that caused swelling elsewhere, such as in the face from *“kidney disease”*. In contrast to the biomedical division into two forms, patients reported four distinct types of podoconiosis. These are presented below using direct translations from Amharic:

1. *”bursts without trauma”*, often reported as *“feminine leg disease” [F3*,*5]*
2. “*Swells without any pain and is soft” [F2*,*4]*
3. *“Swells when touched by something hot” [F1*,*1]*
4. Swells and is *“hard*, *with nodules”* [7]

It was unclear how the different categorisations of disease affected a person’s health-seeking behaviour or self-care practices.

#### Models of causation

Participants offered explanatory models within four broad categories: physical contamination, mystical, divine and genetic, although these categories sometimes overlapped. Former IOCC patients commonly cited contamination with soil as causing their disease, although this was frequently framed with in the notion of the soil being *“cursed”*. Contamination by affected individuals, such as through bathwater or sharing shoes, was widely feared: “*I am sure that it transmits by washing [with] the leftover water from the infected ones” [F2*,*5]*. To prevent this, patients even avoided contact with family members at meals or when bathing.

Mystical causation was frequently ascribed to treading on spells, commonly a “*a spell of an egg, lemon and hen [meat]”* [3], which are cast onto roads by *“diviner-wizards”* or *“Debtra”* (people with an informal church education). Such spells were often created for someone suffering from podoconiosis *“in order to transfer their disease to others”* [9]. For this reason, they could also be used malevolently *“to attack their enemies”* [3].

Divine causation was mainly attributed to punishment for immoral acts, typically not attending church or being undevout. In the absence of any other explanation, participants looked to *“Godly intervention”* as an overarching cause. It was also noted that participants recognised the interaction of heritability with other causes above described as “*triggers”*:

I have said before though [podoconiosis] is already with us as heredity, it needs something as a cause. For example, it may be stepping on magic.”

*[F3*,*5]*

### Traditional treatment

Most patients had sought non-biomedical treatment for podoconiosis and these came in four forms: herbal, magical, folk remedies and faith-based healing.

Herbal treatments were often prepared by diviner-wizards who guarded their secrets closely. Treatment most commonly involved tying a poultice to the leg, which *“created a wound that weeps” [F3*,*5]*. The herbs applied such treatment were: *“Zigba” (Podocorpus falcatus)*[27], *“Demakese” (Ocimum lamiifolum)*[27] and an unidentified plant *“Gishila”*. Other herbal treatment involved boiling the leaves and inhaling the vapours to provoke diaphoresis, including: *“Harage Resa” (Zehneria scabra)*[27] and an unidentified herb named “*Shingung”* in Amharic. Some herbs were unavailable during the dry season, and participants reported concerns about addiction and withdrawal symptoms, especially with *Shingung*. In addition to advising on which herbs to use, certain diviner-wizards were also able to perform magical spells and rites in order to relieve some symptoms of podoconiosis. In some cases, the diviner-wizards would cast the herbs involved in a patient’s treatment into the road as part of a spell.

Folk treatments involved tying fertilizer (a mixture of human urine, soil and water) to a patient’s leg and leaving them to heal. This treatment was very expensive (500-600birr/£15–18) and in some cases aggravated the patient’s symptoms. Other practices included cleansing the legs with lemon and salt water, tying the skin of a dead snake to the affected person’s legs or through lay surgical techniques such that *“the swollen leg is penetrated by blade then the blood is collected by a cup*.*” [F2*,*2]*

Faith-based healing involved washing or drinking holy water or applying holy soil into the legs. Although there are many holy water sites in Northern Ethiopia known for their curative benefits, water could be rendered holy if prayed over by a priest in church, or if the source was the *“result of a miracle” [K1]*. Every patient participant had visited holy water treatment sites and believed them to provide valuable treatment and psychological support with their illness: *“There is holy water, and it relieves. I have visited many holy waters”* [6].

Participants were very amenable to discussing traditional treatments. Perhaps unsurprisingly (given that interviews were conducted in biomedical treatment centres) the majority expressed negative views towards traditional treatments, with the exception of holy water. This is succinctly summarised by one participant:

“Neither the magic spell nor traditional medicine can bring a change.”

[2]

### Health-seeking behaviours

Podoconiosis patients tended to wait until their symptoms were severe before attending services. This appeared to be due to a belief that their symptoms would remit or that visiting a clinic would waste valuable working time for key activities, such as childcare: *“Its only when I fall and am unable to feed my children that I come to the health center*. *Unless I am troubled by the disease*, *I don’t come to health centers*.*” [F1*,*5]*. Only a minority of participants suggested it was better to visit a clinic early in the stages of the disease.

Decisions to attend were predominantly made by men, however, it was stated that women could lead on healthcare access *“if the female is educated” [F2*,*5]*. Traditional forms of treatment were highly valued in the community. Farmers and other members of the community “*inform where the people who give the traditional treatment are found and encourage to go there”* [8]. It was apparent that there is an inherent distrust of modern treatment services, with some participants expressing that such services were *“too poisonous” [F3*,*5]*, ineffective or not modern enough. A fundamental issue was that community members did not believe that the disease “*can be treated simply by washing”* [8]. In one case, a participant stated her fear of clinical errors arising from IOCC treatment because the *“government [could] put me to death by putting the wrong ointment on my leg”* [6]. A few participants expressed that their decision to come to the clinic was due to advice from IOCC workers. Generally, it was after the failure of one particular treatment ‘regime’ that participants sought different options. These tended to be other forms of traditional treatment, though when traditional options were exhausted, Western medical clinics were a last resort.

Participants with no previous contact with IOCC services mentioned that they were not even aware of modern services and when advised about these services responded with: *“Wow! What a pleasant news it would be! If there I will go”* [10].

### Self-care practices

Self-care practices were attributed to valuing wellbeing and “*keeping oneself tidy”* [4]. Participants stated that wellness and general hygiene concepts were taught by the church, whereas specific podoconiosis-related self-care practices were only delivered by the IOCC clinics. Participants expressed that they only really learnt about the importance of washing their legs through observing *“the difference that I feel when I wash and when I don’t wash”* [4]; in some instances, this was ‘discovered’ before attendance at any health-care services.

The most commonly cited reason for failing to adhere to self-care practices was “laziness”. This appeared to amount to exhaustion following long-working days: *“When we arrive home from work*, *we feel tired and we prefer to directly go to bed [rather than wash]” [F3*,*7]*. In addition, collecting water was extremely difficult for those suffering from podoconiosis due to extreme pain when walking. During the dry seasons in Ethiopia, streams and wells could dry up and many patients understandably “*prefer to save the water for drinking than for washing” [F3*,*2]*. Some were able to purchase water in local shops, though *“the time we are living in is difficult”* [11] due to this expense. For the lucky few with access to hand-pumps in their village, they tended to avoid public places due to fear of and past experience of stigma and abuse directed at them.

## Discussion

This qualitative study explored three poorly researched aspects of podoconiosis patients’ health seeking behaviour: explanatory models, use of non-biomedical services and self-care. The findings reveal diverse health beliefs used to account for podoconiosis, pluralistic treatment seeking behaviour and significant barriers to effective self-care. This underlines the importance of these three factors to the health-seeking trajectories of podoconiosis patients. It also suggests that they are closely linked. That is because traditional health beliefs often led participants to pursue interventions other than preventive use of footwear or regular washing and contributed to delayed healthcare attendance and self-care. Belief in divine retribution was reported not only to lead patients to seeking faith-based therapy but could also to engender a sense of the futility of treatment. This study adds to existing knowledge about practical challenges facing podoconiosis patients by highlighting fear within families of contagion, unreported lay treatments and practical barriers to accessing water. A new finding is lay subtypes of podoconiosis that appear to be broader and more nuanced than biomedical categories. Furthermore, gender appeared to play no role in the type of responses given in the study.

Like other studies from Africa,[28] our findings indicate that patients tend to turn to western treatment only when symptoms are particularly severe, and typically after trying a range of traditional treatments. This pluralistic approach is reported elsewhere and is very common.[29] Traditional medicines appeared to be based upon the following principles: cleansing, which could be spiritual (holy water) or physical (lemon juice), releasing fluids (lay surgery or herbal treatments causing wounds/diaphoresis) and faith/symbolism (holy soil, spells). This study uncovered a new faith-based treatment: holy soil. This finding was reported by multiple participants. While infrequent exposure is unlikely to have long-term effects on health, this observation indicates potential ambiguity over the place of soil in the causation and treatment of podoconiosis. Washing with holy water, conversely, is broadly in accordance with the biomedical regime of maintaining foot hygiene and should, therefore, be encouraged on a more frequent amongst patients. Visits to holy water present an untapped resource: with such a high throughput of patients, priests could signpost patients to IOCC services. In addition, there are frequent gatherings in the communities surrounding these holy water sites; promoting discussion of IOCC services, especially highlighting how services are free of charge, could engage a large proportion of podoconiosis sufferers. Expert patients have previously been proposed by Tsegay et al. as a means to shaping community expectations surrounding treatment,[15] and could be similarly utilised in community discussions to promote the services, and adherence to such regimes, offered by the IOCC, as well as a novel method to tackle community-based stigma. This could be a very cost-effective solution to expanding access to services and prompt sufferers to engage with IOCC clinics earlier in the course of their disease.

Unlike many studies in sub-Saharan Africa,[30] most participants expressed negative views about traditional medicines. There may be many reasons for this, beyond that these treatments can be painful and that the interviews were conducted by a researcher affiliated with the treatment centre. Future research should endeavour to explore ways to engage with traditional healers, to learn from their knowledge and promote safety. Again, expert patients could be ideally placed to open this discussion. This is consistent with other calls for greater integration of traditional medicine into the national health service of Ethiopia.[31] A study in 1990 demonstrated that Ethiopian traditional healers were open to this type of co-operation,[32] however, the majority of modern medical practitioners in Ethiopia would prefer not to integrate modern medicine with traditional medicine.[33] Working to overcome this “*cultural-ideological clash”* [34] will be difficult, but grassroots discussion and co-operation is the first step.

This study highlights the key role of water access in promoting self-care. During the dry season (Oct-Mar) rivers and streams that are usually a stable source of water dry up and people are forced to travel further distances to water sources. Most patients neglected washing as any water gathered during this period was essential for survival and used for drinking. Some patients are lucky enough to live in a village with a hand-pump. However, podoconiosis patients often avoid the pump so as to not attract any unwanted attention or stigma from other users. With around 8 litres per day recommended to soak, wash and rinse feet; it is immediately clear that either an alternative to this quantity of water must be found, or that better and more stable water sources are needed for podoconiosis patients. The IOCC and other podoconiosis charities should collaborate with organisations, such as the Ethiopian government’s One WASH National Programme (OWNP), aimed at providing water in order to ensure that wells and pumps are built in locations where need is greatest, and that access to these areas does not provoke further stigma. The economic argument for such an intervention is also strong; with, on average, 45% of total working days lost per year amongst podoconiosis patients,[9] providing water to wash and reduce symptoms will greatly enhance economic productivity.

### Conclusion

The results of this qualitative study indicate the importance of explanatory models, pluralistic health-seeking behaviour and self-care practice in treatment access and care for podoconiosis. It also highlights the interlinked nature of these factors and the need for culturally relevant strategies to improve awareness of, and engagement with, treatment services. This calls for greater understanding and co-operation with priests and traditional healers, including exploration of potential roles in signposting to treatment centres. As reported elsewhere, a further way to engage with lay health beliefs, tackle community stigma and promote access to IOCC services could be through “expert patients”.[15] This study underlines significant challenges to self-care, especially in collecting adequate water for washing. Expanding access to clean water is of utmost importance for the effective treatment of affected persons. Collaboration with OWNP would help water to be available in convenient locations, free of charge and to be collected in such a way as to not promote stigma.
